# MC^3^: a steady-state model and constraint consistency checker for biochemical networks

**DOI:** 10.1186/1752-0509-7-129

**Published:** 2013-11-21

**Authors:** Mona Yousofshahi, Ehsan Ullah, Russell Stern, Soha Hassoun

**Affiliations:** 1 Department of Computer Science, Tufts University, 161 College Ave, Medford, MA 02155, USA

**Keywords:** Constraint-based modeling, Pathway analysis, Model validation, Consistency checking, Model reuse

## Abstract

**Background:**

Stoichiometric models provide a structural framework for analyzing steady-state cellular behavior. Models are developed either through augmentations of existing models or more recently through automatic reconstruction tools. There is currently no standardized practice or method for validating the properties of a model before placing it in the public domain. Considerable effort is often required to understand a model’s inconsistencies before its reuse within new research efforts.

**Results:**

We present a review of common issues in stoichiometric models typically uncovered during pathway analysis and constraint-based optimization, and we detail succinct and efficient ways to find them. We present MC^3^, *Model and Constraint Consistency Checker*, a computational tool that can be used for two purposes: (a) identifying potential connectivity and topological issues for a given stoichiometric matrix, *S,* and (b) flagging issues that arise during constraint-based optimization. The MC^3^ tool includes three distinct checking components. The first examines the results of computing the basis for the null space for *Sv* = 0*;* the second uses connectivity analysis; and the third utilizes Flux Variability Analysis. MC^3^ takes as input a stoichiometric matrix and flux constraints, and generates a report summarizing issues.

**Conclusions:**

We report the results of applying MC^3^ to published models for several systems including *Escherichia coli*, an adipocyte cell, a Chinese Hamster Ovary cell, and *Leishmania major*. Several issues with no prior documentation are identified. MC^3^ provides a standalone MATLAB-based comprehensive tool for model validation, a task currently performed either ad hoc or implemented in part within other computational tools.

## Background

Stoichiometric models play a fundamental role in the analysis and optimization of biochemical networks in Systems Biology and Metabolic Engineering, especially in the absence of detailed kinetic models. A stoichiometric model specifies the relative quantities of reactants and products for each reaction within a network. There are two fundamental and commonly used computational techniques that utilize stoichiometric models to elucidate steady-state function. The first technique is Elementary Flux Mode (EFM) analysis [1,2], used to find all non-decomposable pathways that can operate in steady state. EFM analysis has been used to analyze robustness and regulation [1,3,4], analyze microbial stress responses [5], increase product yield [6], and assess plant fitness and agricultural productivity [7]. Another fundamental technique is Constraint-Based Modeling (CBM), used to analyze flux flow in metabolic networks. CBM encompass several techniques including, Flux Balance Analysis (FBA) [8,9], and Flux-Variability Analysis (FVA) [10]. CBM has recently been extended and applied in innovative ways. Examples include performing whole-genome simulation combining FBA with kinetic rate expressions (dFBA) [11], determining an optimal set of gene modifications with the goal of increasing the production of desired target metabolites [12,13], analyzing genotype–phenotype relationship [14], and performing thermodynamic feasibility analysis [15].

Stoichiometric structural models are traditionally constructed manually, based on earlier models and in combination with reaction availability from databases. An example is the formulation of successive models of the *E. coli* organism that lead to improved predictive capabilities and elucidation of phenotypic behavior [16]. The recent availability of genome, reaction, and organism specific databases have allowed for the automatic reconstruction of genome-scale models. A protocol encompassing 94 steps details the process including obtaining a draft construction from databases, collecting experimental data, refining the reconstruction by adding details to ensure that the network is mass and charge balanced and that missing reactions steps are properly flagged, to test the ability of the model to grow, and to compare against known properties [17]. The tool, Model SEED, expedites this process by automating most of the steps; however, manual curation is still needed to refine the constructed model [18]. The quality of the reconstruction, whether obtained manually or automatically, is as comprehensive as the availability of reconstruction and experimental data, and is a function of the reconstruction procedure. The resulting models may thus be incomplete or inconsistent for the purpose of steady-state analysis. Models can be updated once new information (e.g. genome annotation, reaction directionality) or more accurate reconstruction tools become available.

This paper addresses identifying model inconsistencies in the context of steady-state analysis. Anecdotes within the community show that models released in the public domain often have undocumented inconsistencies, such as dead-end metabolites or reactions incapable of carrying fluxes. While some model issues have been documented [19], there is currently no standalone computational tool that ensures model and constraint consistency. Each user is thus forced to personally validate a model, sometimes in ad hoc and incomplete manners. Alternatively, the user may remain unaware of model issues as some tools work around such issues. For example, in EFMTool [20], a tool for computing Elementary Modes, dead-end metabolites and fluxes that carry a zero flux are removed from the network prior to EFM computation, as part of network compression to speed the EFM computation. Frequently, it is an incorrect computational result that alerts the user to model inconsistencies. In addition to the lack of standalone computational tools for model validation, there is currently no standard documentation protocol that each model undergoes prior to public release. Ideally, each model should provide clear documentation on potential model inconsistency a user might encounter when performing common steady-state analysis tasks such as EFM analysis or CBM. End users would benefit tremendously from a standardized way of identifying and documenting model issues.

We provide in this paper a detailed survey of issues common in stoichiometric models. Each type of issue is identified and explained, and current mechanisms for validating each are reported. The main contribution of this article is to succinctly clarify in one centralized document how each model or constraint property can be most efficiently identified. This paper can be of great benefit to users that wish to implement their own model and constraint consistency checks. We also describe a software tool that we developed, *Model & Constraint Consistency Checker* (MC^3^), which performs model and constraint consistency checking. The MATLAB code that implements this tool is available freely through the web.

## Methods

### Modeling of biochemical networks

**Figure 1:**
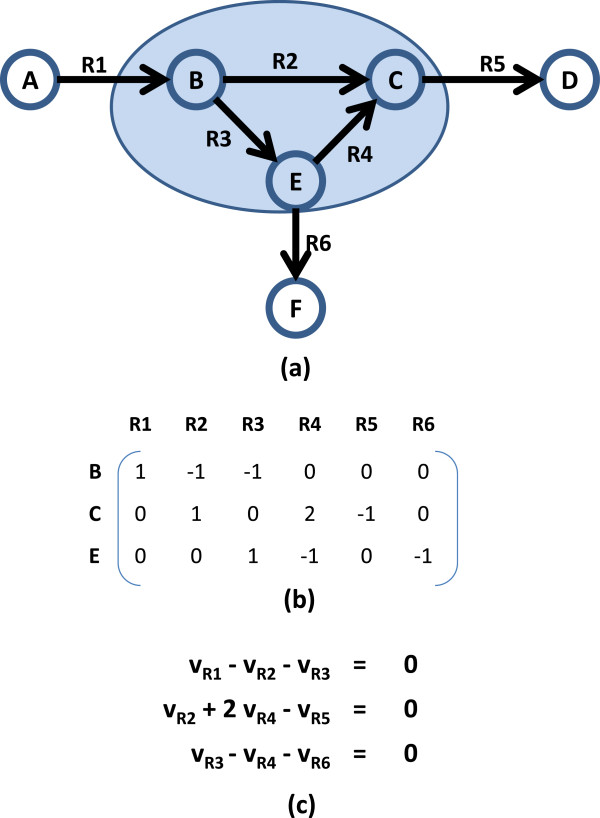
**Example biochemical network. (a)** R1, R5, and R6 represent exchange reactions, and A, D, and F represent external metabolites. **(b)** The corresponding stoichiometric matrix. **(c)** Mass-balance constraints under steady-state conditions.

A biochemical network is represented using a stoichiometric matrix *S *[21,22]. If a network has *m* compounds and *n* reactions, the corresponding matrix *S* will be an *m* by *n* matrix. An entry in the matrix represents the stoichiometric coefficient of a compound participating in a particular reaction. Each column describes a reaction. A column entry is zero if the compound does not participate in the reaction, positive if the compound is a product and negative if the compound is a reactant. Reactions in a network can be classified as exchange or internal reactions. An *exchange* reaction links a biochemical network to its external environment, as defined by the user, and provides either uptake and/or production of external metabolites. Exchange reactions are also referred to as external boundary conditions. Non-exchange reactions are referred to as *internal* reactions that connect internal metabolites. Reactions can be reversible, and are sometimes split into forward and reverse reactions during steady-state and flux balance analysis. Each row in *S* specifies the mass balance relationship for a particular metabolite. During steady-state analysis, external metabolites are excluded from the *S* matrix, while exchange reactions are included. In the example in Figure 1a, there are three internal compounds (B, C and E), three external compounds (A, D and F), and six reactions (R1, R2, R3, R4, R5 and R6). The compound ordering in the stoichiometric matrix in Figure 1b corresponds to B, C, and E; external compounds are not included in the matrix; exchange reactions are R1, R5, and R6.

During steady-state analysis, each internal metabolite in the network is produced and consumed at the same net rate, so that there is no accumulation of internal metabolites. Therefore, when each row in *S*, which describes how each reaction balances a particular metabolite, multiplies *v*, the steady state flux vector, the result must be equal to a zero vector, indicating a zero metabolite accumulation or depletion under steady-state conditions. This relationship is expressed as:

(1) $$\sum_{j=1}^{n}S_{\mathrm{ij}}*v_{j}=0,i\in \left\{1,..,m\right\},$$

where *S*_*ij*_ is the entry in the *i*^*th*^ row and the *j*^*th*^ column in *S.* Thus, at steady state:

(2) Sv=0

Any *v* that satisfies this equation is in the null space (kernel) of *S*. The mass-balance constraints for the example in Figure 1a are illustrated in Figure 1c.

### Computing flux balance and flux variability analysis

Flux Balance Analysis (FBA) is a computational approach to find the flux through the network by solving *Sv* = 0. Typically, the *S* matrix is underdetermined, and many solutions satisfy the steady-state condition. An objective function is typically added to this constraint. FBA is commonly formulated as a linear optimization problem, where the objective function either maximizes or minimizes a desired reaction flux or a combination of desired fluxes, subject to Eq. 2. Additional constraints are used to bound flux ranges. Example constraints are ones that limit uptake or secretion of compounds through exchange reactions, or ones that model the effects of knockouts, and up/down regulation. Users can specify the stoichiometric matrix, the objective function, and the desired bounds in generic linear optimization programs such as GNU Linear Programming Kit (GLPK) and linprog (a linear optimization toolbox within MATLAB), or with a tool such as the COBRA Toolbox [23]. Within the COBRA Toolbox, the default upper and lower bounds are [-1000 1000] for reversible reactions, and [0 1000] for irreversible ones. Flux Variability Analysis (FVA) consists of identifying the minimum and maximum range for each flux subject to steady state condition (Eq. 2) and other model constraints such as uptake or exchange rates. While calculating the minimum and the maximum flux values for each reaction, all external boundaries are set to 0 or -1000 (depending on their reversibility) and 1000. These bounds are reasonable considering typical stoichiometric coefficients of metabolic networks.

### Common model issues

**Figure 2:**
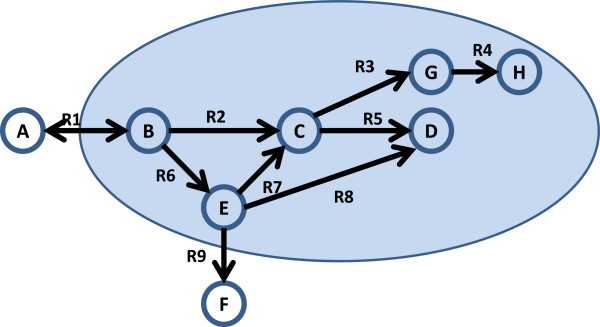
**Illustration of common model issues.** Metabolites D and H are dead-end metabolites as they are only being produced and not consumed. Reactions R2, R3, R4, R5, R7 and R8 are identified by FVA as zero-flux reactions. R1 is an unsatisfied reversible reaction and can carry flux in only one direction. R5 and R8 form a reversibly coupled reaction pair.

Several model and constraint consistency issues arise in practice. The most common is when the model has a dead-end metabolite. There are two existing definitions in the literature for a dead-end metabolite. One definition, termed herein as Singly Connected Metabolite (SCM), specifies internal metabolites with only one participating reaction as a dead-end metabolite [24]. Another definition, termed Dead-End Metabolite (DEM), is when a metabolite internal to the network is either consumed or produced, but not both [20,25]. Both definitions are illustrated in Figure 2. Using definition SCM, H is identified as a singly ended metabolite. Using DEM, both H and D are dead-end metabolites. H is clearly a dead-end metabolite as it will be accumulating during the steady-state operation of the network. If the reaction directionality is correctly specified, and the model has a gap, then D will also accumulate during steady-state operation. Indeed, a non-zero flux value when producing H (through R4) or producing D (through R5 and R8) will violate steady-state constraints imposed by Eq. 1 and Eq. 2. If the directionality of R5 or R8 is specified incorrectly, then alerting the user that D is a dead-end metabolite will help the user correct the directionality of R5 or R8, if appropriate. In either case, the user should be alerted that the metabolites have some connectivity issues, and he/she can then determine the correct course of action. Both SCM and DEM conditions are detected by examining *S,* as follows:

#### *Condition SCM. Singly connected metabolite*

A metabolite *i* is has a single connection *iff*

$$\left|\left\{S_{\mathrm{ij}}|\forall j,\;S_{\mathrm{ij}}\neq 0\right\}\right|=1$$

where {*S*_*ij*_| ∀*j, S*_*ij*_ ≠ 0} refers to the set of non-zero entries in the row associated with metabolite *i*, and the vertical bars refer to the cardinality (number of elements) in that set.

#### *Condition DEM. Dead-end metabolite*

A metabolite *i* is a dead-end metabolite *iff*

$$\forall j:S_{\mathrm{ij}}\geq 0\vee \forall j:S_{\mathrm{ij}}\leq 0,j\in \left\{1,\ldots ,m\right\}$$

where the symbol ∨ corresponds to a logical OR operation.

A second common issue is when a network contains a reaction (r_i_) that can only carry a zero flux for any possible steady-state condition. Several reasons may cause this issue, including r_i_ directly connecting to a dead-end metabolite or to another zero-flux reaction, or if the reversibility of the reaction is specified incorrectly. Burgard et al. refer to zero-flux reactions as blocked reactions [4]. A subset of zero-flux reactions can be detected by analyzing the basis of the null space to ensure that each row has at least one non-zero entry [20]. This method identifies zero-flux reactions due to a connection to a dead-end metabolite, (e.g. R4 is connected to H in Figure 2). However, the null-space basis will not identify R2, R5, R7 and R8 as zero-flux reactions because the null-space basis computation is oblivious to the ability of a reaction to carry flux *only* in a particular direction. FVA, on the other hand, is capable of identifying all zero-flux reactions in the network. The set of zero-flux reactions is identified by maximizing and minimizing the flux of each reaction subject to the network stoichiometry and some bounds [20]. If the maximum and minimum flux of a reaction is zero, then it is a zero-flux reaction. The zero-flux reactions detected by FVA are a superset of the zero-flux reactions identified from the null-space basis vectors. There is thus only need to use FVA and not both FVA and the basis vectors to identify zero-flux reactions. In our tool, we utilize FVA to identify zero-flux reactions using the following condition:

#### *Condition ZFR. Zero-flux reaction*

A reaction *r*_*j*_ is a zero-flux reaction *iff*

$$\left(v_{j\max}=0\right)\wedge \left(v_{j\min}=0\right)$$

where the symbol ∧ corresponds to a logical AND operation, *v*_*j*max_ refers to the maximum flux value for reaction *r*_*j*_ obtained using FVA, and *v*_*j*min_ refers to the minimum flux value for reaction *r*_*j*_ obtained using FVA.

A third common issue that can be detected by inspecting FVA results is an unsatisfied reversibility condition associated with a reversible reaction. Sometimes reactions marked as reversible carry flux only in one direction. Terzer uses LP-based feasibility analysis to find unsatisfied reversible reactions [20]. An unsatisfied reversibility condition was found if the minimum or maximum flux value is zero for a reversible reaction. This definition, however, excludes reactions that have a non-zero positive minimum or a non-zero negative maximum from consideration. Here, we expand the definition of unsatisfied reversibility to additionally include reversible reactions that can carry only positive non-zero or only negative non-zero flux. Once identified, reactions identified as having unsatisfied reversibility can be marked if appropriate, as only-forward or only-backward based on the signs of their fluxes. We detect unsatisfied reversibility using the following condition:

#### *Condition UR. unsatisfied reversibility*

A reversible reaction *r*_*j*_ has unsatisfiable reversibility *iff*

$$\left(v_{j\max}\leq 0\right)\vee \left(v_{j\text{min}}\geq 0\right)$$

where the symbol ∨ corresponds to logical OR operation. Some zero-flux reversible reactions can also be declared as zero-flux reactions.

Another steady-state characteristic of interest is flux coupling among a pair or group of irreversible reactions. Such pairs or groups have been referred to as “enzyme subsets” [26] and they were shown to have similar expression patterns, share transcriptional regulators, and frequently reside in the same operon [27]. Knowledge of coupled reactions enables finding equivalent knockouts and, when used in conjunction with directionality data, enables the identification of missing reactions in a reconstruction [4]. If the fluxes of two reactions are always constant multiples of each other, then the reactions are coupled. If additionally the constant multiplier is negative, then the reaction pair is labeled as reversibly coupled. Such conditions can be detected by checking the null-space basis matrix and examining the coupling ratios [20,26]. We detect coupling using the following conditions.

#### *Condition CR. coupled reactions*

Two irreversible reactions, *r*_*i*_ and *r*_*j*_*,* are coupled *iff*

$$\forall b_{i*},b_{j*}\;\text{pairs},\frac{b_{i1}}{b_{j1}}=\frac{b_{i2}}{b_{j2}}=\cdots =\frac{b_{\mathrm{ip}}}{b_{\mathrm{jp}}}$$

where *b*_i*_ is a row in the null-space basis of the *S* matrix associated with a reaction *i*, *b*_*ik*_ and *b*_*jk*_ are either both zero, or both non-zero entries in the *k*^*th*^ column in the null-space basis matrix, and *p* is the number of columns in the basis matrix.

#### *Condition RCR. reversibly coupled reactions*

Two irreversible reactions, *r*_*i*_ and *r*_*j*_*,* are reversibly coupled *iff*

$$\forall b_{i*},b_{j*}\;\text{pairs},\frac{b_{i1}}{b_{j1}}=\frac{b_{i2}}{b_{j2}}=\cdots =\frac{b_{\mathrm{ip}}}{b_{\mathrm{jp}}}<0$$

The set of reactions with condition RCR is a subset of the reactions in CR.

### MC^3^ implementation

Despite the availability of literature coverage of some of the issues described in the previous section, there is clearly a need to develop a computational tool to perform model and constraint consistency checking in a comprehensive, and standalone way. Such a tool enables each user to check his or her model before releasing it to others to ensure that constraints are consistent and the model can be exercised as desired. A user may exercise the tool upon receiving a model from another user or when obtaining a model from a database or supplementary material. Additionally, such a tool can be utilized as a front-end to other tools that utilize stoichiometric models. To this end, we describe a software tool that we developed, the *Model & Constraint Consistency Checker* (MC^3^) tool. As mentioned earlier, MC^3^ code is implemented using MATLAB [28]. However, before running MC^3^, two software dependencies, namely the SBML toolbox [29] and GLPK [30] need to be installed.

**Figure 3:**
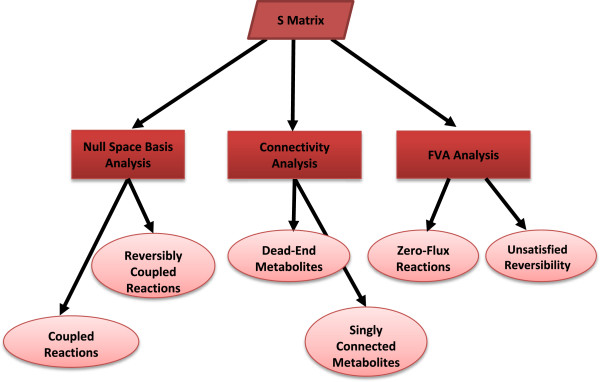
**MC**^**3 **^**tool overview.**

Figure 3 provides an overview of MC^3^. Initially, the *S* matrix is read. Three types of computations (in boxes) are used: null-space basis analysis, connectivity analysis, and FVA analysis. Each computation checks the appropriate conditions marked in the ovals.

The main module of MC^3^ is mc_checkmodel. It reads the model and runs the checks specified by its checkType parameter. The first parameter of mc_checkmodel is the model type, which can either be ‘xls’ or ‘SBML’ [31] depending on the model type. The second parameter is checkType which can be 0, 1, or 2, where 0 corresponds to running only checks based on the null-space basis vectors (Conditions CR, RCR), 1 corresponds to running only checks based on FVA (Condition ZFR and UR), and 2 corresponds to running checks using both. Conditions SCM and DEM are always checked. The third parameter is the filename. If the file type is ‘xls’, then the remaining parameters are the Excel sheets for the stoichiometry, exchange reactions, external metabolites, and user-defined bounds. The *S* matrix is formatted with rows as metabolites and columns as reactions. The last row specifies reaction reversibility (1 for reversible, and 0 for irreversible). Every cell should have a numerical entry. The exchange reactions are expressed as a single column. Each entry contains the index of an exchange reaction. The external metabolites are also expressed as a single column. Each entry contains a row number of the relevant metabolite. The *S* matrix is stripped of all external metabolites before analysis. The external metabolite listing is therefore optional. The lower and upper bound arrays for FVA analysis are expressed as two columns. The first is the lower bound array and the second column is the upper bound array. There must be one row for each reaction in the *S* matrix and each entry within the row must contain a numerical value. The flux bounds are optional. If not specified, MC^3^ uses the upper and lower bounds [-1000 1000] for reversible reactions, and [0 1000] for irreversible ones. If the file type is ‘SBML’, then the remaining parameters are arrays containing the exchange reactions, external metabolites, and user-defined bounds. The exchange reactions are expressed as a single column. Each entry contains a reaction ID. The external metabolites are also expressed as a single column. Each entry contains a metabolite ID. The bonds are specified as they were for the xls’ case. All three parameters are optional.

An example of running mc_checkmodel with all checks for the SBML formatted model (Ec_iAF1260_flux1) [32] is provided. The function is run as follows:

[SCM, DEM, ZFR, UR, CR, RCR] = mc_checkmodel (‘SBML’, 2, ‘Ec_iAF1260_flux1.xml’, ‘ExchangeReactions’, ‘ExternalMetabolites’, ‘OverrideUserBounds’);

When the command finishes executing, the return elements (SCM, DEM … ) are arrays that contain indices of either metabolites or reactions that pertain to that check, or will be empty if that check did not return any results. The indices can be correlated with the SBML file by examining the struct that holds the *S* matrix, and reaction and metabolite names. The example model, Ec_iAF1260_flux1, results in the report shown below.

Statistics:

2382 reactions

852 reversible reactions

299 exchange reactions

1668 metabolites

Connectivity Checks:

87 Singly connected metabolites (SCM)

118 dead-end metabolites (DEM)

Basis-based checks:

970 coupled reactions (CR)

45 reversibly coupled reactions (RCR)

FVA-based checks:

184 zero-flux reactions (ZFR)

420 unsatisfied reversibility (UR)

The report has four sections. The first reports various statistics associated with the network and ensures that the whole network was read in. The second section reports the results of connectivity analysis. The third section reports the results from the analysis that used the basis vectors. In the last section, we report the results of FVA-based analysis.

We have compared the functionality of MC^3^ with other available tools, namely, the COBRA toolbox [23], CellNetAnalyzer [33] and MetaNetX [34,35]. While dead-end metabolites can be determined using all these tools, zero-flux reactions are only reported using MC^3^, CellNetAnalyzer and MetaNetX. Both MC^3^ and MetaNetX can find coupled reactions. All other discussed issues can only be identified using MC^3^. The comparison is summarized in Table 1.

**Table 1:** Summary of tool comparison

	**MC3**	**COBRA**	**CellNetAnalyzer**	**MetaNetX**
**Singly connected metabolite**	●			
**Dead-end metabolite**	●	●	●	●
**Zero-flux reaction**	●		●	●
**Unsatisfied reversibility**	●			
**Coupled reactions**	●			●
**Reversibly coupled reactions**	●			

'●' refers to the presence of a functionality.

## Results and discussion

We checked the consistency of some available models using MC^3^. The checked models included those of *E.coli* (3 different sizes) [6,25,32], adipocyte [36], Chinese Hamster Ovary (CHO) cell [37], and *L. major *[38]. For every test case, MC^3^ checks for all conditions specified in section ‘Common Model issues’. A summary of the results is shown in Table 2.

**Table 2:** **Summary of applying MC**^**3 **^**to several published models**

**Model**	**(a) Metabolites**	**(b) Reactions**	**(c) Exchange reactions**	**(d) Reversible reactions**	**(e) SCM**	**(f) DEM**	**(g) ZFR**	**(h) UR**	**(i) CR**	**(j) RCR**
Minimal *E. Coli*	53	70	15	19	2	2	3	8	14	0
*E. Coli* iJR904	761	1075	143	388	56	67	150	196	325	8
*E. Coli* iAF1260	1668	2382	299	852	87	118	184	461	970	32
adipocyte	26	34	7	0	0	0	0	0	10	0
CHO cell	231	275	34	108	0	0	0	53	540	0
*Leishmania major*	1101	1112	64	630	250	259	376	374	156	9

For each model, the number of metabolites, reactions, exchange reactions and reversible reactions are indicated in columns a-d. The results of applying MC^3^ are indicated in columns: (e) Singly Connected Metabolites (SCM), (f) Dead-End Metabolites (DEM), (g) Zero-Flux Reactions (ZFR), (h) Unsatisfied Reversibility (UR), (i) Coupled Reactions (CR), and (j) Reversibly Coupled Reactions (RCR).

The *E. coli* central metabolic network [6], composed of 70 reactions and 53 internal metabolites (52 internal metabolites as reported in the paper [6] plus BIOMASS), captures the conversion of sugars to ethanol. For this network, MC^3^ reports two dead-end metabolite (BIOMASS and ATP_main) and three related zero-flux reactions. In the supplementary section of the paper [6], it is noted that metabolite names containing ‘ext’ are external metabolites. BIOMASS was thus treated as an internal metabolite. BIOMASS and ATP_main each appear only in one reaction. Both BIOMASS and ATP_main are thus identified by MC^3^ as dead-end metabolites. Reactions directly connected to these metabolites are identified as zero-flux reactions.

*E. coli* model *i*JR904 [25] contains 70 dead-end metabolites that are listed in the additional data file. These metabolites participate in 89 reactions that will never be engaged if the network operates at steady state. The reactions that trace back to the dead-end metabolites in *i*JR904 are purposefully included for later amendments when more annotation data is available. MC^3^ identifies 67 dead-end metabolites and 150 zero-flux reactions in this network. The difference in the number of dead-end metabolites compared to the reported results is due to metabolites cardiolipin, lipopolysaccharide, and peptidoglycan. Each participates as a reactant in the biomass reaction.

*E. coli* model iAF1260 [32] is a reconstructed model based on *i*J904R. The model documentation (Table 2) reports 304 exchange reactions, while the published model has only 299 exchange reactions. The authors state clearly that there are some dead-end metabolites within the model but do not provide specific documentation. MC^3^ identifies 118 dead-end metabolites and 184 zero-flux reactions based on the FVA computation. The model documentation reports using thermodynamic feasibility and flux variability analysis to adjust the model based on assessing reaction reversibilities; however, we identified 461 out of 852 reversible reactions that still had unsatisfied reversibilities.

The Adipocyte model is a small network with 26 metabolites and 34 reactions [36]. This model does not have any of the discussed issues.

The CHO cell model capturing the central carbon metabolism is part of a larger network [37]. The reversibility of reactions in this model is derived from the KEGG database, and reaction directions are also derived from KEGG. MC^3^, however, identifies 53 reactions that operate only in one direction.

For the *L. major* model [38], 261 dead-end metabolites are reported. MC^3^ is able to find 259 dead-end metabolites and 376 zero-flux reactions based on the FBA computation, which include the reactions connected directly to the dead-end metabolites or connected to the other zero-flux reactions. There are 374 unsatisfied reversible reactions.

It is important to realize that our tool (and others) can only validate a *sub*set of inconsistencies associated with a particular model. Further, the tool validates properties of the model, and not the correctness of the model or the reconstruction. For example, MC^3^ identifies the conditions for dead-end metabolites. The end user is to decide how to interpret this result -- it can be due to an incomplete reconstruction or perhaps an incorrect sign in the file specifying the model. Another example is specifying incorrectly the coefficients of a particular reaction. The issue of *intent* vs. *specification* exists in verification and validation of other engineering and software systems. MC^3^ cannot verify the intent, but it can certainly validate some aspects (properties) of the model. Validation of biological models will become more prominent in this field with its maturity, and with the increase use of automated reconstruction tools.

## Conclusions

While model and consistency constraint issues have been identified in various contexts, this article offers a review and detailed methodology for checking common issues. All checks are packaged within MC^3^, a tool available for others to validate their model. The results of applying MC^3^ to several models have found some inconsistencies between the models and their respective published articles. It would be important for the community to have a standard method of documenting and reporting issues with each published models. The MC^3^ MATLAB files are available by contacting the authors or through GitHub at https://github.com/sohahassoun/mc3.

## Abbreviations

EFM: Elementary flux mode; CBM: Constraint-based modeling; FBA: Flux balance analysis; FVA: Flux-variability analysis; MC3: Model & constraint consistency checker; GLPK: GNU linear programming kit; SCM: Singly connected metabolite; DEM: Dead-end metabolite; ZFR: Zero flux reactions; UR: Unsatisfied reversibility; CR: Coupled reactions; RCR: Reversibly coupled reactions; CHO: Chinese hamster ovary.

## Competing interests

The authors declare that they have no competing interests.

## Authors’ contributions

SH conceived the idea. MY, EU, and RS wrote and documented the code. MY and EU ran the test cases. Everyone contributed to the writing of the manuscript. SH and MY revised the document. All authors read and approved the final manuscript.
